# Supplementary material for: Refractive plasma optics for relativistic laser beams
Source: Nat Commun. 2023 Jun 6;14:3296. doi: 10.1038/s41467-023-38937-0 (PMC10244328; doi:10.1038/s41467-023-38937-0)
Supplement: Supplementary file 1 — Supplementary Information [file 41467_2023_38937_MOESM1_ESM.pdf]

Supplementary Information for Refractive plasma optics for relativistic laser beams

Supplementary Note 1.

In here we present consecutive shot data depicting the beam pointing (Supplementary Fig. 1), energy spectrum data for the case with heater beam (Supplementary Fig. 2) and with astigmatism correction (Supplementary Fig. 3)

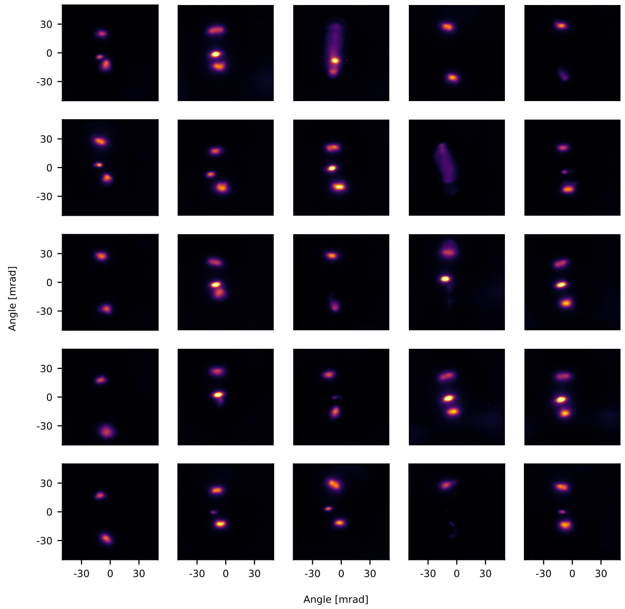

Supplementary Fig. 1 Consecutive beam profile measurements in experiments with heater beam depicting stability of the multi-beam phenomenon and beam pointing variance.

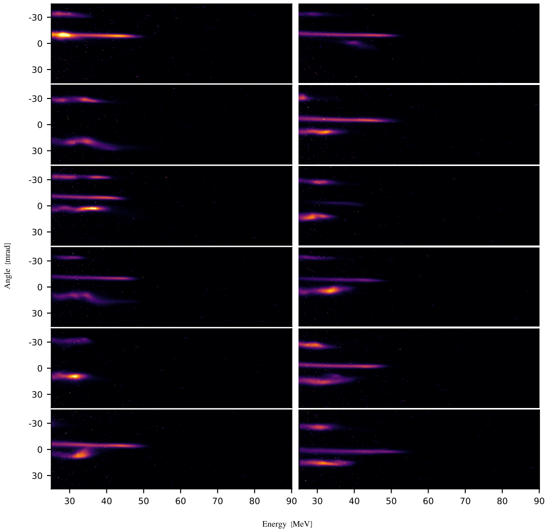

Supplementary Fig. 2 Consecutive energy spectrum measurements in experiments with heater beam.

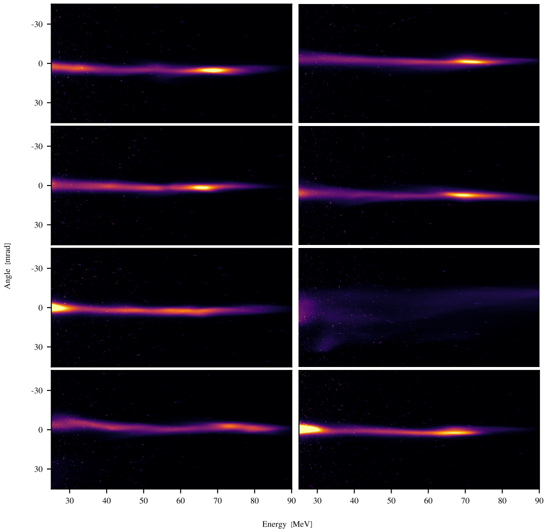

Supplementary Fig. 3 Consecutive energy spectrum measurements in experiments with heater beam and with astigmatism correction.

Supplementary Note 2.

In here we indicate the vacuum evolution of the laser beam with the astigmatism correction used in experiment. In the Supplementary Fig. 4, the intensity of the astigmatic beam have factor 3 gain compared to the pictures without astigmatism. The full angle divergence of this astigmatism is approximately 50 mrad in the near field.

During the experiment, the positive astigmatism was added through the adaptive optics to correct the phase of the main laser, and it can be understood as pulling back the vertical focus by about 500 μm and meanwhile pushing forward the horizontal focus with the same amount (see the upper part in Supplementary Fig. 4). By adding this term, the laser pulse was supposed to diverge vertically after the vertical focal position, and this compensates the later focusing effect from the vertical gradient of the plasma structure in the low-density region. By carefully fine-tuning the amounts of the added astigmatism, we could adjust the vertical and horizontal focus of the laser in the plasma back to roughly the same position. Consequently, a single electron bunch with much better beam quality (i.e., charge and maximum energy) compared to the null case was produced. In fact, it is also possible to reach the above condition by adding certain amounts of negative astigmatism term together with a shifting of the focus term. This focus shift however was not tested during our experiment due to the increased complexity, and experiments with added negative astigmatism saw much decreasing electron beam parameters.

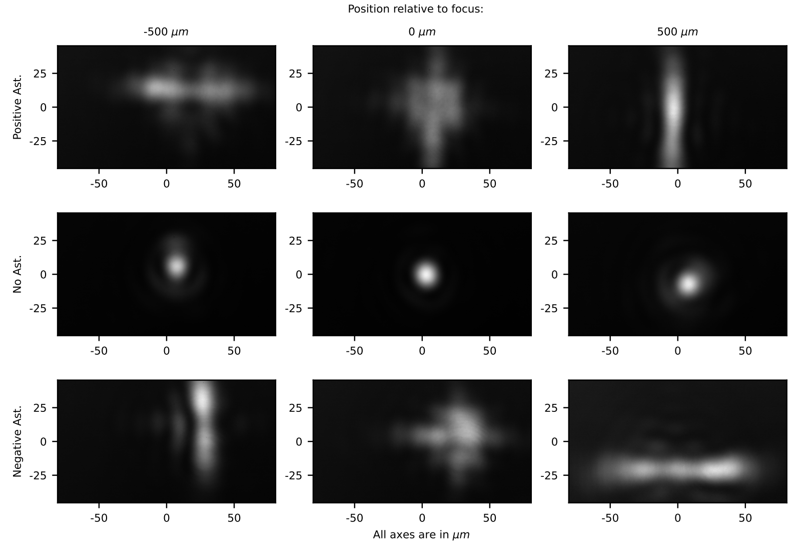

Supplementary Fig. 4 Vacuum focal spot evolution for three cases of the positive astigmatism (up), no astigmatism (middle) and negative astigmatism (down) respectively (all axes are in μm). The no astigmatism case, for which we see multiple beams when heater beam is introduced, has a much cleaner evolution throughout the target compared with the astigmatic cases.
